# Exosomal ALPPL2 and THBS2 as biomarkers for early detection and disease monitoring of pancreatic ductal adenocarcinoma

**DOI:** 10.1038/s41416-025-03167-2

**Published:** 2025-09-03

**Authors:** Kuntal Halder, Erkut H. Borazanci, Gayle S. Jameson, Wei Lin, Amber Vrana, Derek Cridebring, Susan Tsai, Mohammed Aldakkak, Douglas B. Evans, Masamichi Hayashi, Haruyoshi Tanaka, Mitsuro Kanda, Ajay Goel, Daniel D. Von Hoff, Haiyong Han

**Affiliations:** 1 Clinical Genomics and Therapeutics Division, Translational Genomics Research Institute, Part of City of Hope, Phoenix, AZ USA; 2 HonorHealth Research Institute, Scottsdale, AZ USA; 3 Department of Surgery, The Medical College of Wisconsin, Milwaukee, WI USA; 4 Department of Gastroenterological Surgery, Nagoya University Graduate School of Medicine, Nagoya, Japan; 5 Department of Molecular Diagnostics and Experimental Therapeutics, Beckman Research Institute, City of Hope Comprehensive Cancer Center, Duarte, CA USA; 6 Present Address: Ohio State University Comprehensive Cancer Center, Columbus, OH USA

**Keywords:** Tumour biomarkers, Pancreatic cancer

## Abstract

**Background:**

Lack of reliable biomarkers for early detection and monitoring contributes to the poor prognosis of pancreatic ductal adenocarcinoma (PDAC), as the current clinical marker, CA19-9, lacks adequate specificity and sensitivity.

**Methods:**

Serum concentrations of ALPPL2-positive and THBS2-positive exosomes were measured using an ExoView assay in two cohorts: a cohort of 219 subjects, including non-disease controls and patients with early- or late-stage PDAC, and a longitudinal cohort of 26 patients with advanced PDAC undergoing treatment.

**Results:**

Exosomal ALPPL2 and THBS2 distinguished non-cancer cases from PDAC with high accuracy; area under the curve (AUC) values = 0.983, 0.993, and 0.983 for ALPPL2, THBS2, and the dual marker combination, respectively. Additionally, changes in the concentrations of ALPPL2^+^ and THBS2^+^ exosomes strongly correlated with radiographic tumor size changes during treatment in both CA19-9-elevated (*p* = 0.016 and 0.014 for ALPPL2 and THBS2, respectively) and non-elevated patients (*p* = 0.003 and 0.006 for ALPPL2 and THBS2, respectively).

**Conclusions:**

Serum exosomal ALPPL2 and THBS2 can accurately discriminate patients with PDAC from individuals with non-cancerous conditions and healthy controls. Changes in serum exosomal ALPPL2 and THBS2 levels significantly correlate with patients’ response to treatment in both CA19-9-elevated and non-elevated patients.

## Background

Pancreatic cancer is currently the 3^rd^ leading cause of cancer related death in the United States [1]. Despite advances in our understanding of the biology of pancreatic cancer, progress in early detection and effective treatment of the disease has been limited [2, 3]. Patients with pancreatic ductal adenocarcinoma (PDAC), which is the most common type of pancreatic cancer, often present with non-specific symptoms, leading to late-stage diagnosis. At the time of diagnosis, only 15–20% of the patients are eligible for surgical resection, the primary treatment offering potential long-term survival [4, 5]. One study reported a 5-year survival rate of 17.4% for patients with resectable PDAC, compared to just 0.9% for those with unresectable disease [6]. In a prospective Phase II study, Tsai and colleagues showed that patients with resectable or borderline resectable PDAC had a median survival of 45 months after neoadjuvant therapy and surgery compared to 11 months for those who underwent neoadjuvant therapy but not surgery [7]. Therefore, the development of new diagnostic tools, particularly non-invasive methods, is not just critical but urgent for improving early detection and outcomes for patients with PDAC.

Currently, no reliable, non-invasive test exists for accurate early-stage detection of PDAC. The limitations of current imaging methods and the diagnostic utility of the carbohydrate antigen 19-9 (CA19-9) underscore the necessity for new, more effective diagnostic tools. Imaging methods such as computerized tomography (CT), magnetic resonance imaging (MRI), and endoscopic ultrasonography are expensive and have low sensitivity and specificity for detection of small malignant lesions [8]. CA19-9 which is the FDA-approved serum biomarker for PDAC is primarily used for disease monitoring and prognosis. Its diagnostic utility is limited by poor sensitivity, false negatives in Lewis antigen-negative individuals, and false positives from benign or malignant biliary diseases [9, 10]. While the search for new, non-invasive biomarkers with superior performance to CA19-9 continues, very few have been adequately tested or validated for clinical implementation [11, 12].

In recent years, exosomes, small membranous vesicles ranging from 30 to 150 nm in size, have emerged as a promising source of biomarkers for cancer [13]. These vesicles contain molecular cargos, including proteins, lipids, and nucleic acids, from their cells of origin and are thought to be critical mediators of cell-to-cell communication [14]. Importantly, cancer cells, including those from pancreatic tumors, secrete exosomes that carry unique pathogenic signatures distinct from those of normal cells [15]. Exosomes derived from PDAC tumor tissues or cells have been shown to contain specific proteins and RNA molecules detectable in systemic circulation [16, 17]. These tumor-derived molecular cargos represent a valuable opportunity for developing non-invasive diagnostic tools for the early detection of PDAC. Investigators are actively exploring the profiling of exosomal content, including RNA signatures, proteomic patterns, and lipidomic profiles, to identify highly specific and sensitive markers for PDAC [18]. As such, the study of exosome-based biomarkers represents a promising technique for the early detection of PDAC. Their potential to transform early detection strategies and improve outcomes for patients with this aggressive malignancy is a cause for optimism in the field of oncology and molecular diagnostics.

In our search for exosome-based protein markers for PDAC, we measured the levels of a panel of proteins in circulating exosomes isolated from serum samples of patients with pancreatic cancer. These proteins were previously reported to be overexpressed in pancreatic tumor tissues or cells. We found that two proteins, alkaline phosphatase, placental-like 2 (ALPPL2) and Thrombospondin 2 (THBS2), were highly elevated in serum exosomes isolated from patients with PDAC compared to those from healthy controls. ALPPL2, also known as alkaline phosphatase, germ cell type (ALPG), is a glycosyl phosphatidyl inositol (GPI)-anchored membrane protein that belongs to the alkaline phosphatase (ALP) isozyme family [19]. ALPPL2 has been reported to be overexpressed in multiple tumor types such as mesothelioma [20], testicular [21], ovarian [22], gastric [23], and pancreatic cancer [24]. THBS2 is a member of the calcium-associated glycoprotein family and is also overexpressed in multiple cancers including PDAC [25–27]. Here, we report the utility of circulating exosome-based ALPPL2 and THBS2 as biomarkers for PDAC. We demonstrate that the concentrations of ALPPL2^+^ and THBS2^+^ exosomes are significantly elevated in the serum samples of patients with PDAC compared to healthy controls and to patients with non-cancerous conditions such as pancreatitis and cystic lesions. Changes in the concentrations of these marker positive exosomes significantly correlated with response (changes in tumor size) to treatment in patients with advanced PDAC.

## Methods

### Samples and patient populations

De-identified serum samples from two patient cohorts were used in this study. The first cohort (*N* = 219) included patients diagnosed with pancreatitis, intraductal papillary mucinous neoplasms (IPMNs), mucinous cystic neoplasm (MCN), pancreatic intraepithelial neoplasia (PanIN), or PDAC at different stages (Stage I, II, III, and IV) as well as healthy controls (defined as no prior benign pancreatic conditions or cancer diagnosis at the time of sample collection) that were enrolled in biospecimen collection programs at the Translational Genomics Research Institute (TGen), City of Hope, HonorHealth Research Institute, Medical College of Wisconsin, and Nagoya University School of Medicine. The demographics and clinical characteristics of the cohort are summarized in Table 1 (Data for individual patients are provided in Supplementary Table S1). The second cohort (*N* = 26) included patients with advanced metastatic PDAC treated at HonorHealth Research Institute. Longitudinal serum samples at regular intervals (1–2 months) were collected from those patients and provided to this study. The patient demographics for the second cohort are shown in Table 2. This study was conducted in accordance with the Declaration of Helsinki. The collection of the samples was approved by the institutional review boards of the institutions. A written informed consent was obtained from each of the subjects. Serum samples were aliquoted and stored at −80 °C until analysis. PDAC patients with CA19-9 levels ≥37 U/mL were classified as having elevated CA19-9, while those with levels <37 U/mL were considered non-elevated or normal.

**Table 1:** Demographics of early detection cohort including subjects with different diagnoses.

Demographics		Healthy control (HC)	Non-cancerous conditions (NCC)			PDAC			
			Pancreatitis	Cystic neoplasm	PanIN	Stage			
						I	II	III	IV
*N*		57	6	41	2	30	24	17	42
Age (Y)	<60	24	3	8	0	4	6	3	4
	≥60	33	3	33	2	26	18	14	38
Gender	Male	19	3	20	1	14	18	8	21
	Female	38	3	21	1	16	6	9	21

**Table 2:** Demographics of PDAC disease monitoring cohort including both CA19-9-elevated and non-elevated patients.

Demographics		PDAC (Stage IV)	
		CA19-9 elevated	CA19-9 non-elevated
*N*		16	10
Age (Y)	<60	5	6
	≥60	11	4
Gender	Male	12	10
	Female	4	0

### Western blotting analysis of exosomal proteins

Exosomes were isolated using the SmartSEC^TM^ Single kit (System Biosciences, Palo Alto, CA, USA) by following the manufacturer’s instructions. Exosomes were lysed with 1× RIPA buffer (Cell Signaling Technology, Danvers, MA, USA). The separation, transfer, and probing of exosomal proteins (10 µg/sample) were carried out as described previously [28]. The primary antibodies against ALPPL2 (Cat #H00000251-M07, Abnova Corporation, Taiwan), THBS2 (Cat #GTX636411, GeneTex, Irvine, CA, USA), CD81 (Cat #sc-166029, Santa Cruz Biotechnology, Santa Cruz, CA, USA), and GAPDH (Cat #sc-137179, Santa Cruz Biotechnology) were incubated with membranes overnight at 4 °C. Protein bands were detected using chemiluminescence and imaged using an Odyssey DLx Imaging System (LICORbio, Lincoln, NE, USA). The images were further analyzed to quantify the protein band intensities using the ImageJ software [29].

### Measurement of marker positive-exosome concentrations in serum samples using ExoView

Serum samples were centrifuged at 2000 × *g* for 10 min and 10,000 × *g* for 30 min at 4 °C to thoroughly remove large and small cellular debris [30]. Samples were further filtered through a 0.2 μm pore size filter to exclude particles > 200 nm in diameter.

The measurement of ALPPL2 and THBS2 positive exosome concentrations in the serum samples was carried out by using the EV-TETRA-C Exosome Detection Kit on an ExoView R100 platform (Unchained Labs, Pleasanton, CA, USA). The ExoView platform allows for the characterization of antigen (e.g., tetraspanins) captured exosomes using interferometric (IM) imaging and immunofluorescence staining at single vesicle level [31]. We used microchips that were precoated with antibodies against three tetraspanins, CD9, CD81, and CD63 to capture exosomes and then stained them with fluorescently labeled antibodies against ALPPL2 and THBS2 to detect the marker-positive exosomes. The antibodies were the same as those used in the Western blotting and labeled with CF^®^-555 (anti-ALPPL2) and CF^®^-488 (anti-THBS2) using the Mix-n-Stain™ CF® Dye Antibody Labeling Kit (Biotium, CA, USA). The buffer solutions (Incubation Solution, Blocking Buffer, and Solution A, B, C, and D) were included in the kit. Microchips coated with exosome-capturing antibodies against three tetraspanin proteins (CD9, CD63, and CD81) were pre-scanned to obtain baseline measurements. Serum samples (40 µL) diluted in Incubation Solution were carefully loaded onto the microchips and incubated in a sealed 24-well plate at room temperature overnight. On the next day, microchips were submerged in 1000 µL of Solution A and incubated for 5 min. Subsequently, 750 µL of Solution A was removed, replaced with 750 µL of fresh Solution A and incubated for 5 min. This washing step was repeated three times. After the third wash, 250 µL of Solution C was added to the chips and incubated for 10 min followed by 3 washings with 750 µL of Solution A. To permeabilize the exosomes, 250 µL of Solution D was added to the chips and incubated for 10 min followed by 3 washings with 750 µL of Solution A. For non-permeabilized conditions, the incubation step with Solution D was skipped. In parallel, antibody detection mixtures were prepared as a two-fold concentrate by diluting fluorescently labeled antibodies at 1:500 in the Blocking Buffer. After the last washing step, 250 µL of the antibody mixture was added to the microchips and incubated for 1 h at room temperature. The microchips were washed with 750 µl of Solution A three times as described above followed by an additional three washes with 750 µL of Solution B and one wash with 750 µL of ultrapure water. The microchips were placed on lint-free paper wipes to allow the remaining water to drain and then imaged with the ExoView R100 fluorescent and interferometric imager (Unchained Labs). The chip images were analyzed using the ExoView Analyzer software (Unchained Labs) to obtain the marker-positive exosome concentrations for each tetraspanin capture. The results are reported as the mean measurement of three independent biological replicates and their respective technical triplicates ± standard error of the mean, unless stated otherwise. Graphs were generated using GraphPad Prism software (Version 10, GraphPad Software, Boston, MA, USA).

In the experiment to assess the specificity of the ExoView assay in detecting exosomes, serum samples were pretreated with Triton X-100 (final concentration 5%, v/v) for 1 h at room temperature before being used in the ExoView assay as described above.

### Measurement of markers in human serum samples using ELISA

To measure the soluble ALPPL2 and THBS2, enzyme-linked immunosorbent assays (ELISAs) were carried out using commercial kits. The ALPPL2 (Cat #DLR-ALPPL2-Hu) and THBS2 (Cat. #DTSP20) ELISA kits were obtained from DLdevelop (Katy, TX, USA) and R&D Systems (Minneapolis, MN, USA), respectively. The assays were carried out by following the manufacturers’ instructions. Serum samples were diluted 8-fold in dilution buffers provided by the kits and run in duplicates. Marker concentrations were determined from the standard curves of positive controls provided by the kits.

### Data statistical analysis

Statistical analyses of the results were performed using the GraphPad Prism software and R (Version 4.4.1). Student’s *t* test (two-tailed) was used to compare differences between the two groups. Fisher’s exact test was used to determine the statistical significance of the correlation between changes in marker-positive exosome concentrations and patient responses. Area under the curve values (AUCs) derived from the receiver operating characteristic (ROC) curves were calculated with 95% confidence intervals using the GraphPad Prism software. A linear mixed model was used to calculate the significance of the association between marker-positive exosome concentration and tumor size in the longitudinal samples. A *P* value of <0.05 was considered statistically significant.

## Results

### Serum exosomal ALPPL2 and THBS2 protein levels are elevated in patients with PDAC

**Fig. 1:**
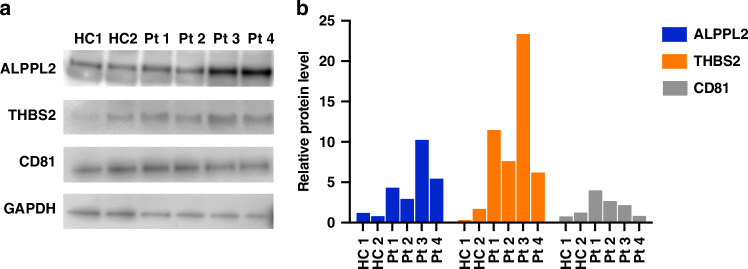
ALPPL2 and THBS2 protein levels in exosomes isolated from serum samples of healthy controls and PDAC patients detected by Western blotting. **a** Western blot images for individual protein markers. CD81 (a tetraspanin protein) was included as a positive control for exosomes. **b** Relative protein levels (normalized to the average of HC samples) based on the quantification of band intensities in (**a**).

To determine if exosomal ALPPL2 and THBS2 protein levels were elevated in serum samples from patients with PDAC compared to those from healthy controls we first used Western blotting to analyze the protein levels in exosomes isolated from the serum samples of four patients with Stage IV PDAC and two healthy controls. As shown in Fig. 1a, ALPPL2 and THBS2 levels in exosomes from patients with PDAC were 3–10 and 6–23 times higher than those in healthy controls, respectively. In comparison, the exosomal marker CD81 showed similar levels between the patients with PDAC and healthy controls (Fig. 1a, b). These results support that serum exosomes from patients with PDAC have higher levels of ALPPL2 and THBS2 proteins than those from healthy controls.

### ExoView assay for measuring ALPPL2^+^ and THBS2^+^ exosome concentrations in serum samples

**Fig. 2:**
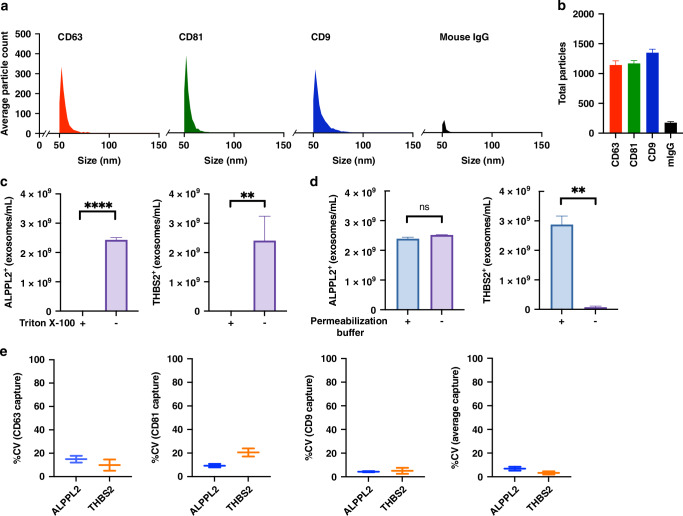
ExoView assay for measuring ALPPL2^+^ and THBS2^+^ exosome concentrations in serum samples. **a** ExoView interferometric (IM) imaging size distribution of exosomes captured by antibodies against different tetraspanins in serum samples from a patient with PDAC. **b** Number of particles (exosomes) captured by antibodies against different tetraspanins in the serum sample from a patient with PDAC based on IM imaging. **c** Effects of detergent (Triton X-100) treatment on ExoView detection of ALPPL2^+^ and THBS2^+^ exosomes in serum samples from patients with PDAC. **d** Effects of permeabilization treatment on ExoView detection of ALPPL2^+^ and THBS2^+^ exosomes in serum samples from patients with PDAC. **e** Inter-assay CV (%) for the ExoView assays stratified by different tetraspanin captures. ****P < 0.0001; **P < 0.01. *N*  ≥ 3 for each group.

To more quantitatively measure the levels of exosomal ALPPL2 and THBS2 proteins in serum samples, we developed an ExoView assay for determining the concentrations of ALPPL2^+^ and THBS2^+^ exosomes. Figure 2a shows the IM (particle) size distribution of captured exosomes in a patient with PDAC. The majority (>95%) of exosomes captured had a diameter between 50 and 75 nanometer (nm) with a median of 52 nm (the minimal diameter size detected by the ExoView R100 interferometric imager is 50 nm). This size distribution is consistent with those reported [31]. The average number of exosome particles captured by the CD9, CD81, and CD63 antibodies was 1087, 1138, and 1310, respectively (Fig. 2b).

To verify that the ALPPL2^+^ and THBS2^+^ signals are from exosomes, we treated the serum sample with detergent (Triton X-100 at 5%, v/v) to lyse the exosomes before incubating with the microchips. As shown in Fig. 2c, treatment with Triton X-100 almost completely abolished the signal for both ALPPL2 and THBS2. Furthermore, to determine if the ALPPL2 and THBS2 molecules detected by the ExoView were on the surface or inside of exosomes we compared the signals with or without the permeabilization buffer treatment. ALPPL2^+^ exosome concentration showed no significant difference with or without permeabilization, whereas the concentration of THBS2^+^ exosomes was reduced by >98% without permeabilization, indicating that ALPPL2 is localized to the surface (or transmembrane) of exosomes whereas THBS2 is mostly intravesicular (Fig. 2d). Based on this result, vesicle permeabilization was applied in all subsequent analysis of patient samples. Finally, to assess the inter-assay consistency of the ExoView assay we repeated the measurement of the same serum sample 3 or more times on different days. As shown in Fig. 2e, the coefficient of variation (CV) between the repeats is between 2 and 18%, indicating an excellent assay performance. Together, these results demonstrate that our ExoView assay for ALPPL2 and THBS2, which uses unprocessed serum samples, is exosome and antigen-specific and highly consistent.

### Exosomal ALPPL2 and THBS2 as biomarkers for early detection of PDAC

**Fig. 3:**
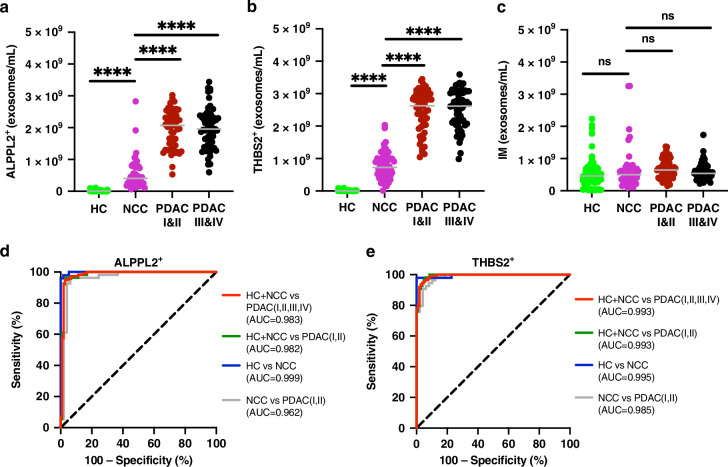
Performances of exosomal ALPPL2 and THBS2 as early detection biomarkers for PDAC. Concentrations of ALPPL2^+^ (**a**), THBS2^+^ (**b**), and interferometric (IM) imaging (**c**) exosomes in serum samples are plotted for individual subjects by groups as listed in Table 1. Concentrations are the average of three tetraspanin captures. ROC analysis was conducted to compare the performances of exosomal ALPPL2 (**d**) and THBS2 (**e**) in discriminating all stages of PDAC from healthy control (HC) and non-cancerous conditions (NCC), early stage (Stage I and II) PDAC from HC and NCC, NCC from HC, and early stage PDAC from NCC. ****P < 0.0001. ns not significant (*P* ≥ 0.05).

To assess whether or not exosomal ALPPL2 and THBS2 in serum can be used as early detection markers for PDAC, using the ExoView assay described above, we measured the concentrations of ALPPL2^+^ and/or THBS2^+^ exosomes in serum samples from a cohort of subjects that included healthy controls (*N* = 57) and patients with non-cancerous conditions (*N* = 49 including 6 pancreatitis and 43 cystic neoplasm or pancreatic intraepithelial neoplasia cases), as well as early stage (Stage I and II, *N* = 54) or late stage (Stage III and IV, *N* = 59) PDAC (Table 1). As shown in Fig. 3a, b, the concentrations of ALPPL2^+^ and THBS2^+^ exosomes calculated as the average of 3 tetraspanin (CD9, CD81, and CD63) captures are significantly (*P* < 0.0001) different among the different subject populations. The healthy controls (HC) had the lowest (mean = 1.4 × 10^7^ exosomes/mL for ALPPL2^+^ and 8.7 × 10^6^ exosomes/mL for THBS2^+^), patients with PDAC had the highest (mean = 2.0 × 10^9^ exosomes/mL for ALPPL2^+^ and 2.5 × 10^9^ exosomes/mL for THBS2^+^), and non-cancerous conditions (NCC) were in the middle (mean = 5.3 × 10^8^ exosomes/mL for ALPPL2^+^ and 7.6 × 10^8^ exosomes/mL for THBS2^+^). However, the number of exosomes captured by the tetraspanin antibodies as measured by interferometric (IM) imaging did not significantly differ among the different subject populations (*P* > 0.05, Fig. 3c). This suggest that the total number of exosomes in the serum samples were very similar among the various subject populations. Similar results were obtained when the marker-positive exosome concentration was analyzed by individual tetraspanin captures (Supplementary Fig. S1). Of note, although they were slightly lower in Stage I patients, the concentrations of ALPPL2 and THBS2 exosomes were not significantly different among different PDAC stages (*P* > 0.05, Supplementary Fig. S2).

Notably, the concentrations of marker-positive exosomes showed a broader range in patients with PDAC or NCC compared to HC (Fig. 3a, b). To evaluate the utility of exosomal ALPPL2 and THBS2 as early detection markers for PDAC, we carried out a ROC analysis to determine the performance of the markers in discriminating between different diagnoses. Figure 3d, e shows the ROC curves of ALPPL2 and THBS2 positive exosomes in discriminating PDAC (all stages) from NCC and HC with an area under the curve (AUC) value of 0.983 (95% CI: 0.945–0.991) and 0.993 (95% CI: 0.957–0.990), respectively. Comparison of other different diagnostic groups also showed similarly impressive AUC values for the exosomal markers, particularly, between NCC and early-stage PDAC which had AUC values of 0.962 (95% CI: 0.920–1.000) and 0.985 (95% CI: 0.968–1.000) for ALPPL2 and THBS2, respectively. We also evaluated the performance of dual ALPPL2 and THBS2 positive exosomes in discriminating different diagnoses. The concentrations of dual ALPPL2^+^/THBS2^+^ exosomes were significantly different between PDAC and control (HC and NCC) samples [AUC = 0.983 (95% CI: 0.952–0.995) for PDAC vs. HC and NCC, Supplementary Fig. S3A–E]. However, dual ALPPL2^+^/THBS2^+^ exosomes did not appear to outperform individual markers based on the ROC analysis (Supplementary Fig. S3B). Overall, these results suggest that exosomal ALPPL2 and THBS2 exhibit high potential as early detection markers for PDAC.

To compare the performances of exosomal ALPPL2 and THBS2 with those of soluble protein levels, we measured the serum ALPPL2 and THBS2 levels using commercially available ELISA kits. As shown in the Supplementary Fig. S4A, B, both serum ALPPL2 and THBS2 showed significant differences between HC, NCC and PDAC. ROC curve analyses also revealed that serum ALPPL2 and THBS2 had decent AUC values of 0.919 and 0.875, respectively, in discriminating control (HC and NCC) from early stage PDAC [HC + NCC vs. PDAC (I&II), Supplementary Fig. S4C, D]. In comparison, the AUC values of exosomal ALPPL2 and THBS2 as single markers in discriminating the case and control subjects are significantly higher at 0.982 and 0.993, respectively. These results provide further evidence for the superior performance of exosomal ALPPL2 and THBS2 and strengthen the potential of their clinical utility.

### Exosomal ALPPL2 and THBS2 as biomarkers for disease monitoring in CA19-9 elevated or non-elevated PDAC

**Fig. 4:**
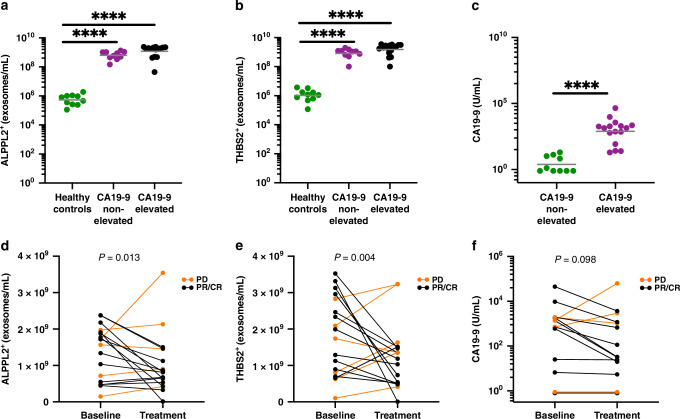
Exosomal ALPPL2 and THBS2 as biomarkers for disease monitoring in patients with PDAC. **a** ALPPL2^+^ exosome concentrations at baseline in CA19-9-elevated and non-elevated patients and age-matched healthy controls (HC). **b** THBS2^+^ exosome concentrations at baseline in CA19-9-elevated and non-elevated patients and age-matched HC. **c** Serum CA19-9 concentrations at baseline in CA19-9-elevated and non-elevated patients (37 U/ml was used as the cutoff) and age-matched HC. **d** ALPPL2^+^ exosome concentrations before treatment and at a timepoint after treatment when the patient showed either a positive radiographic response (partial response, PR, or complete response, CR) or progressed disease (PD) based on RECIST 1.1 criteria. **e** THBS2^+^ exosome concentrations before treatment and at a timepoint after treatment when the patient showed either a PR/CR or PD. **f** Serum CA19-9 concentrations before treatment and at a timepoint after treatment when the patient showed a either a PR/CR or PD. Lines in D-F connect paired samples from one patient. ****P < 0.0001. ns not significant.

To investigate the utility of exosomal ALPPL2 and THBS2 in PDAC disease monitoring, we measured the marker-positive exosome concentrations in longitudinal serum samples obtained from a cohort of patients with PDAC undergoing treatment at HonorHealth Research Institute (Table 2). CA19-9 levels in the serum samples and tumor sizes by the Response Evaluation Criteria in Solid Tumors Version 1.1 (RECIST 1.1) at corresponding time points (within 2 weeks of blood being drawn) were also obtained. Of the 26 patients who had serum samples and tumor size data for at least 3 time points, including the baseline (before treatment initiation), 10 were classified as CA19-9 non-elevated (CA19-9 < 37 U/mL for all time points) and 16 were CA19-9 elevated (CA19-9 ≥ 37 U/mL for at least one of the time points). The ALPPL2^+^ and THBS2^+^ exosome concentrations and the corresponding CA19-9 levels and tumor size measurements for all time points are detailed in Supplementary Table S2. As can be seen in Fig. 4a–c, the concentrations of ALPPL2^+^ and THBS2^+^ exosomes at baseline were similar between CA19-9-elevated and non-elevated PDAC cases and much higher than those in age-matched healthy controls, which is consistent with what was observed for the early detection cohort (Fig. 3). We then evaluated the correlation between changes in ALPPL2^+^ and THBS2^+^ exosome concentrations (average of the 3 tetraspanin captures) before and after treatment and radiographic responses. Figure 4d–f shows the concentrations of ALPPL2^+^ and THBS2+ exosomes as well as CA19-9 levels before treatment (Baseline), and at the time point of response (PR, partial response or CR, complete response) or progressive disease (PD) based on RECIST 1.1 criteria. Patients who had stable disease over the course of treatment were not included in the analysis. Changes in ALPPL2^+^ and THBS2^+^ exosome concentrations were significantly correlated with the radiographic response; *P* values of 0.013 and 0.004, respectively (Fig. 4d–e). In contrast, the changes in CA19-9 levels were not significant (*P* = 0.098) based on Fisher’s exact test (Fig. 4f) as the CA19-9 levels did not change in the majority of the CA19-9 non-elevated patients. We further analyzed the correlation between the changes in ALPPL2^+^ and THBS2^+^ exosome concentration throughout the treatment course with the changes in tumor size using linear mixed models in individual patients grouped by CA19-9 status (elevated, non-elevated, and all subjects). Table 3 shows the *P* values of the correlation analysis based on each tetraspanin capture or the average of the 3 tetraspanin captures. ALPPL2 showed a significant (*P* < 0.05 with the lowest at 0.0002) correlation with tumor size for all 3 tetraspanin captures and the average of the 3 captures for all three groups. THBS2 showed a significant correlation (*P* < 0.05) with tumor size in CD81 capture for all three groups but not for the CD9 and CD63 capture or the average capture (except the average capture in the all subject group, which has a *P* value of 0.02). As a comparison and as expected, the CA19-9 level only showed a significant correlation in the CA19-9 elevated group with a *P* value of 0.045, but not in the non-elevated or all-subject group (*P* > 0.05). Collectively, these data suggest that ALPPL2 and THBS2 could be used to monitor treatment response in patients with PDAC, particularly those with non-elevated CA19-9 levels.

**Table 3:** Correlation between marker positive exosome concentration and tumor size in CA19-9 elevated or non-elevated patients.

Analysis group	# of Patient (N)	*P* value (CD81)		*P* value (CD63)		*P* value (CD9)		*P* value (Average)		CA19-9
		ALPPL2	THBS2	ALPPL2	THBS2	ALPPL2	THBS2	ALPPL2	THBS2	
All subjects	26	0.001	0.001	0.0002	0.063	0.0002	0.115	0.0002	0.02	0.057
CA19-9 elevated	16	0.016	0.014	0.003	0.113	0.002	0.164	0.004	0.054	0.045
CA19-9 non-elevated	10	0.003	0.006	0.013	0.25	0.0143	0.345	0.005	0.092	0.095

## Discussion

In the current study, we demonstrated the potential utility of exosomal ALPPL2 and THBS2 as diagnostic and disease-monitoring biomarkers for PDAC. As mentioned earlier, ALPPL2 has been reported to be overexpressed in multiple tumor types including PDAC [20–24]. ALPPL2 is also present in extracellular vesicles isolated from conditioned media of PDAC cells in culture [32]. Expression of ALPPL2 in normal tissues is generally restricted to placenta [20]. Hence, ALPPL2 has been considered a specific tumor cell surface antigen and a potential therapeutic target [20, 33–35]. Among the four ALP isozymes in humans - the other three members include placental type (ALPP), intestinal type (ALPI), and non-tissue specific type (ALPL), ALPPL2 and ALPP share 98% amino acid sequence homology. Hence, antibodies developed for ALPPL2 are often cross-reactive with ALPP [20]. We tested several commercially available antibodies for ALPPL2, and they all reacted with ALPP including the one used in this study (Supplementary Fig. S5A). Therefore, it is likely that the exosomes identified in the ExoView assays were positive for either ALPPL2 or ALPP or both. ALPP has also been reported to be overexpressed in multiple solid tumor types, including ovarian and testicular germ cell tumors, and endometrial carcinoma [36]. Our analysis of The Cancer Genome Atlas (TCGA) dataset for PDAC (TCGA-PAAD) showed that, similar to ALPPL2, high expression of ALPP mRNA is significantly associated with poor survival (Supplementary Fig. S5B, C). Antibody-drug conjugates targeting both ALPPL2 and ALPP are being developed for treatment of patients with solid cancers, including PDAC [35, 37]. These data indicate that both ALPPL2 and ALPP can serve as potential biomarkers as well as possible therapeutic targets for multiple cancers.

Multiple studies have reported the potential of serum or plasma total THBS2 as a diagnostic marker for PDAC [26, 38–41]. In a Phase II study of healthy controls and patients with PDAC at various stages of disease, plasma THBS2 levels, combined with serum CA19-9, was able to discriminate those with PDAC from healthy controls; AUC value = 0.97 [41]. However, in a subsequent study with prediagnostic (1–15 years prior to a cancer diagnosis) plasma samples from patients with PDAC, the AUC value for the marker pair in discriminating cases from healthy controls was <0.8 [27]. Byrling and colleagues also reported that serum total THBS2 improved the power of CA19-9 in discriminating healthy controls from early-stage PDAC but did not provide additional value to CA19-9 in differentiating benign pancreatic diseases from PDAC [39]. Our results suggest that serum exosome-based THBS2 could be a better biomarker for PDAC, with a higher AUC value, in discriminating not only between healthy control and early-stage PDAC but also between non-cancerous conditions (e.g., IPMN and pancreatitis) and all stages of PDAC, regardless of CA19-9 levels. However, the performance of serum exosomal THBS2 in prediagnostic samples remains to be investigated.

The current study does not address the question of where the marker-positive exosomes are originated. ALPPL2 was reported to be overexpressed in pancreatic cancer cells [24] and THBS2 is mainly produced by stromal cells [42]. Therefore, it is plausible that ALPPL2^+^ exosomes in the serum are mainly originated from pancreatic tumor cells whereas THBS2^+^ exosomes are produced by stromal cells such as cancer-associated fibroblasts. However, additional studies are needed to determine the exact origins of these circulating exosomes.

We observed differences in marker-positive exosome concentrations across the three tetraspanin (CD63, CD81, and CD9) captures (Supplementary Fig. S1). Several factors may contribute to these differences: (1) distinct subpopulations of exosomes express varying levels of tetraspanins—for example, some exosomes may express CD9 but not CD63 or CD81, and vice versa; (2) variations in tetraspanin surface density can affect capture efficiency; and (3) differences in antibody affinity and epitope accessibility may influence binding. These variations do not imply that one tetraspanin is better than another; rather, each capture reflects a different subpopulation of exosomes. Therefore, in our analysis, we used the average concentration from all three captures as the primary measurement.

Radiographic imaging modalities such as CT, PET-CT and MRI are often used to monitor treatment response in patients with PDAC, and are most commonly performed at two- to four-month intervals. Non-invasive, low-cost biomarkers that can be assessed more frequently are highly desirable. CA19-9 may be considered in this category, however, small changes in serum levels may not directly correlate with response or disease progression [43, 44]. Further, ~15–20% of patients with PDAC do not have an elevated level of CA19-9 [45, 46]. CA19-9 production in patients with PDAC also exhibits racial disparities, with blacks being three times more likely to be non-elevated compared to whites [45]. For those with non-elevated levels of CA19-9, carcinoembryonic antigen (CEA) and CA125 are sometimes used to monitor disease progression. However, it has been reported that <40% of CA19-9 negative patients have an elevated level of either CEA or CA125 [46]. Better biomarkers are needed for disease monitoring in patients with PDAC, particularly those who are CA19-9 non-elevated. In recent years, circulating tumor DNA (ctDNA) has emerged as a promising marker for solid cancers including PDAC [47, 48]. ctDNA assays are more frequently used in clinical practice to identify actionable mutations as a guide to treatment selection or to detect minimal residual disease (MRD) after curative surgery [49]. However, the utility of ctDNA as a marker of disease progression and treatment response has not been fully validated. Although ctDNA concentration has been reported to correlate with radiographic tumor burden, the strength of this association is highly variable during longitudinal assessments [50].

Our results demonstrated that ALPPL2^+^ and THBS2^+^ exosome concentrations correlated competently with radiographic tumor burden (tumor size based on RECIST 1.1) in patients with PDAC regardless of their CA19-9 secretion status. These exosome concentrations also perform better than CA19-9 in patients who are CA19-9 non-elevated. Serum exosomal ALPPL2 and THBS2 offer a new biomarker for the early detection of PDAC and have the potential to be used to monitor disease progression and therapy response. One limitation of our study is that samples used were retrospectively collected and the cohort sizes were relatively modest (*n* = 106 for non-disease controls and 113 for PDAC cases for the early detection cohort and *n* = 26 for the disease monitoring cohort). Another limitation of this study is that it did not test the effect of serum storage conditions (e.g., storage temperature and duration) as well as the batch effect for longitudinal samples. Hence, larger validation cohorts are needed to confirm the results. In addition, discriminating high-grade dysplasia/invasive cancer from low-grade dysplasia in patients with worrisome cystic lesions is an unmet clinical need. Due to the small number of cyst cases with known grade information in our early detection cohort, we were not able to test the utility of our markers in this clinical setting.

To further validate the utility of the exosome biomarkers for the early detection of pancreatic cancer, multiple additional studies are needed, including retrospective longitudinal repository studies and prospective screening studies [51]. The retrospective longitudinal studies will evaluate the markers in longitudinal pre- and at-diagnostic samples to define the criteria for a positive screening test including the cutoff value for a marker. The positive screening criteria will be used in subsequent prospective screening studies which will determine the detection rate and the false referral rate in relevant populations [51]. To further verify the utility of the exosomal markers in disease monitoring, prospective longitudinal studies in patients undergoing treatment will be needed, particularly those do not have elevated CA19-9 levels.

## Conclusions

In summary, we have demonstrated that ALPPL2 and THBS2 are elevated in exosomes isolated from serum samples of patients with PDAC compared to healthy controls. Furthermore, serum exosomal ALPPL2 and THBS2 can discriminate patients with PDAC, including early stage diseases (Stage I and II), from individuals with non-cancerous conditions and healthy controls with high accuracy (AUC > 0.980). Changes in serum exosomal ALPPL2 and THBS2 levels significantly correlate with treatment response in both CA19-9 elevated and non-elevated patients (*p* < 0.016). Prospective studies are warranted to further validate the clinical utilities of these markers.

## Supplementary information

Supplementary Information

Supplementary Table S1

Supplementary Table S2

REMARK-checklist-for-EQUATOR

## Data Availability

The RNA transcriptome profiling data and patient outcome data for the TCGA pancreatic adenocarcinoma cohort (TCGA-PAAD) are publicly available in the portal site of TCGA (https://portal.gdc.cancer.gov/projects/ TCGA-PAAD). All other data generated or analyzed during this study are included in this published article and its supplementary information files.
