# Supplementary material for: Exosomal ALPPL2 and THBS2 as biomarkers for early detection and disease monitoring of pancreatic ductal adenocarcinoma
Source: Br J Cancer. 2025 Sep 3;133(9):1335–43. doi: 10.1038/s41416-025-03167-2 (PMC12572646; doi:10.1038/s41416-025-03167-2)
Supplement: Supplementary file 1 — Supplementary Information [file 41416_2025_3167_MOESM1_ESM.docx]

**Supplementary Information**

**
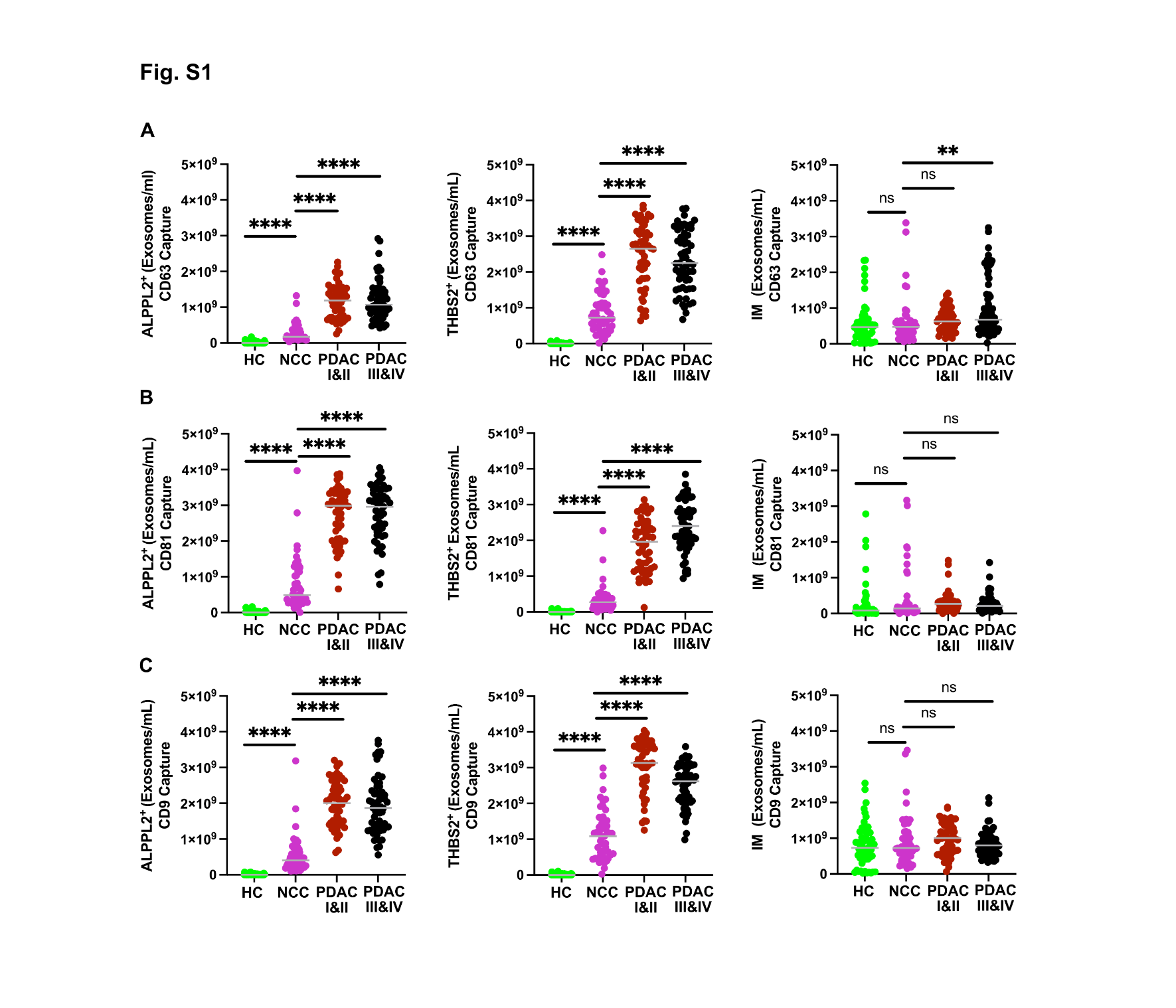
**

**Supplementary Fig. S1**. Dot plots for ALPPL2^+^ and THBS2^+^ exosome concentrations and exosomes captured (IM) in different diagnostic cohorts for individual tetraspanin captures: A) CD63 capture, B) CD81 capture, and C) CD9 capture.

**
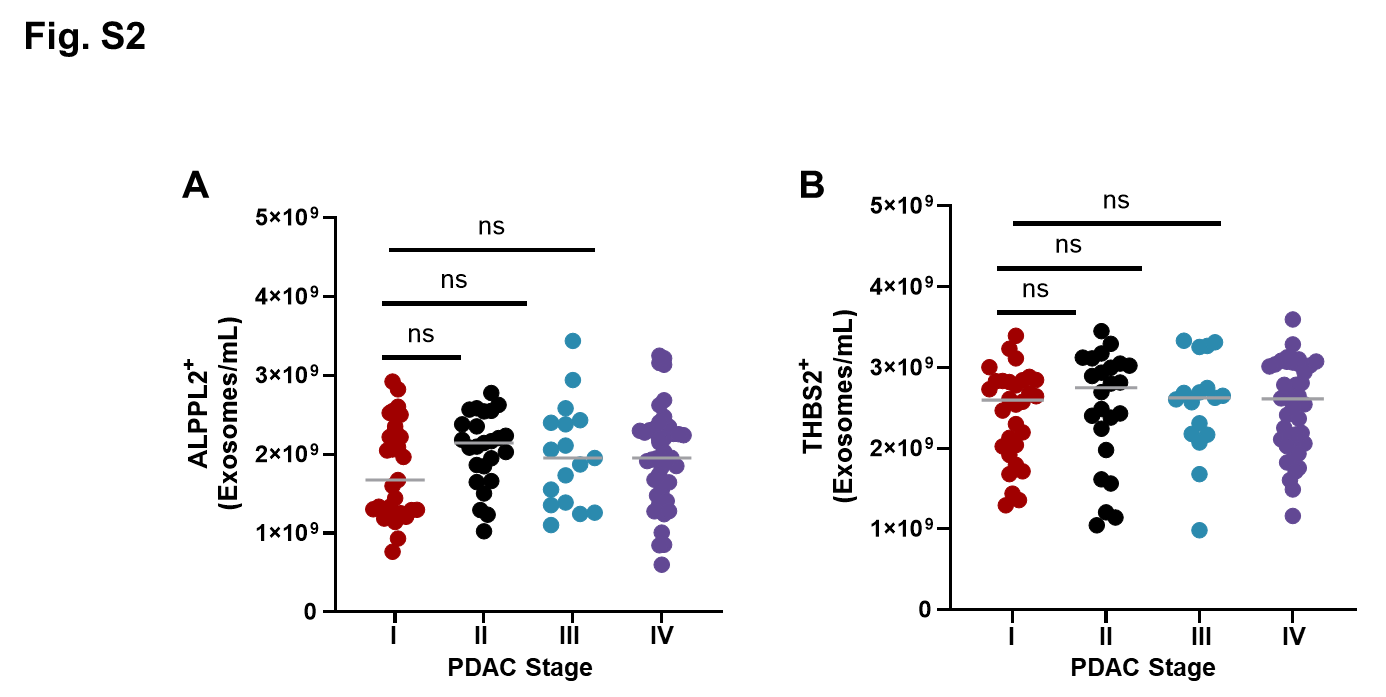
**

**Supplementary Fig. S2**. Dot plots for ALPPL2^+^ (A) and THBS2^+^ (B) exosome concentrations in patients with Stage I, II, III, or IV pancreatic ductal adenocarcinoma (PDAC).

**
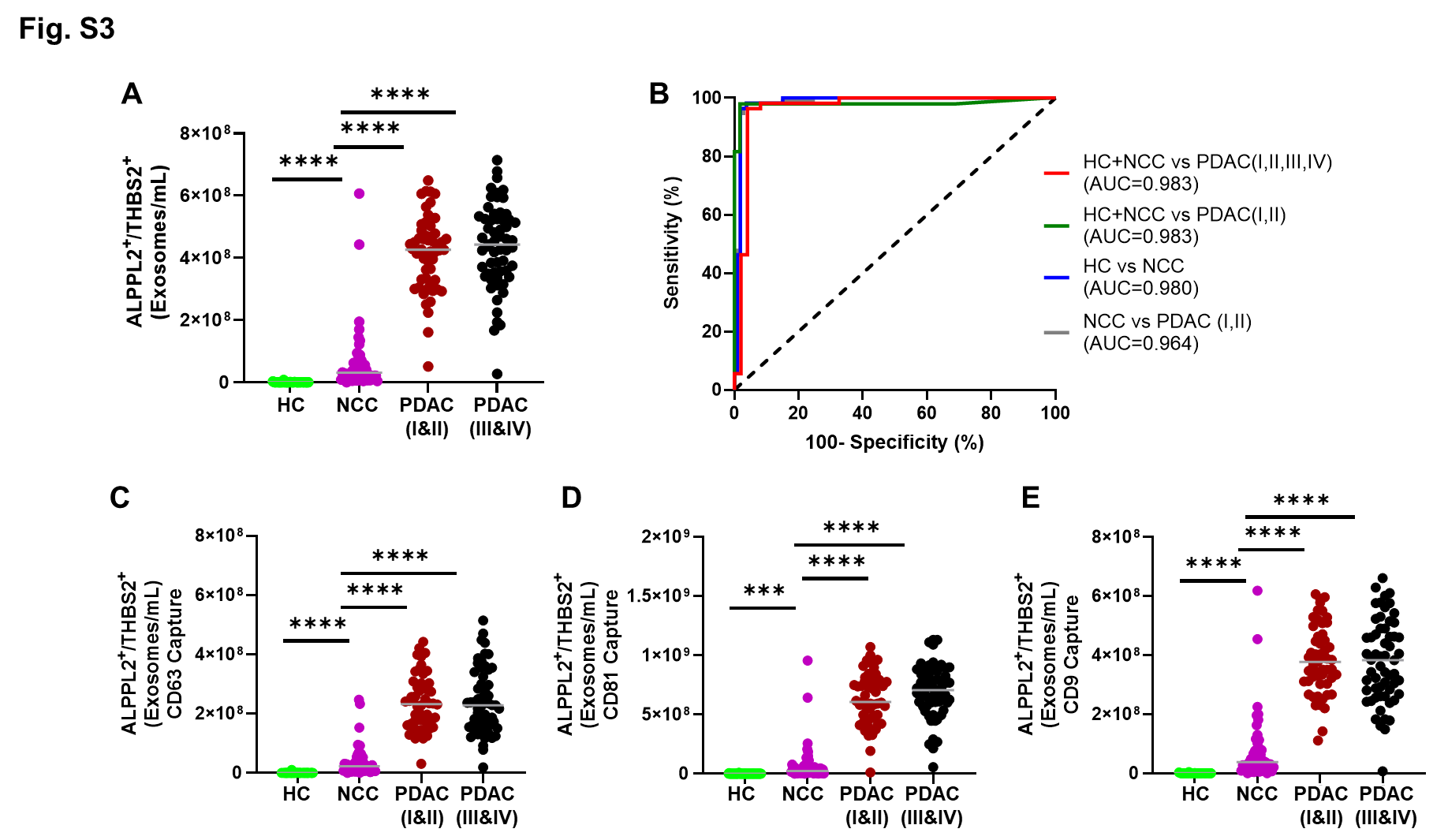
**

**Supplementary Fig. S3**. Performance of dual exosomal ALPPL2 and THBS2 as early detection biomarkers for PDAC. Concentrations of ALPPL2/THBS2 dual positive exosomes are plotted for individual subjects by groups as listed in Table 1. A) Concentrations of dual marker positive exosomes as the average of three tetraspanin captures. B) ROC analysis comparing the performance of dual exosomal ALPPL2/THBS2 biomarkers in discriminating all stages of PDAC from healthy control (HC) and non-cancerous conditions (NCC), early stage (Stage I and II) PDAC from HC and NCC, NCC from HC, and early stage PDAC from NCC. C, D, and E) Concentrations of dual marker positive exosomes as individual tetraspanin captures. ****P < 0.0001. ***P < 0.001.

**
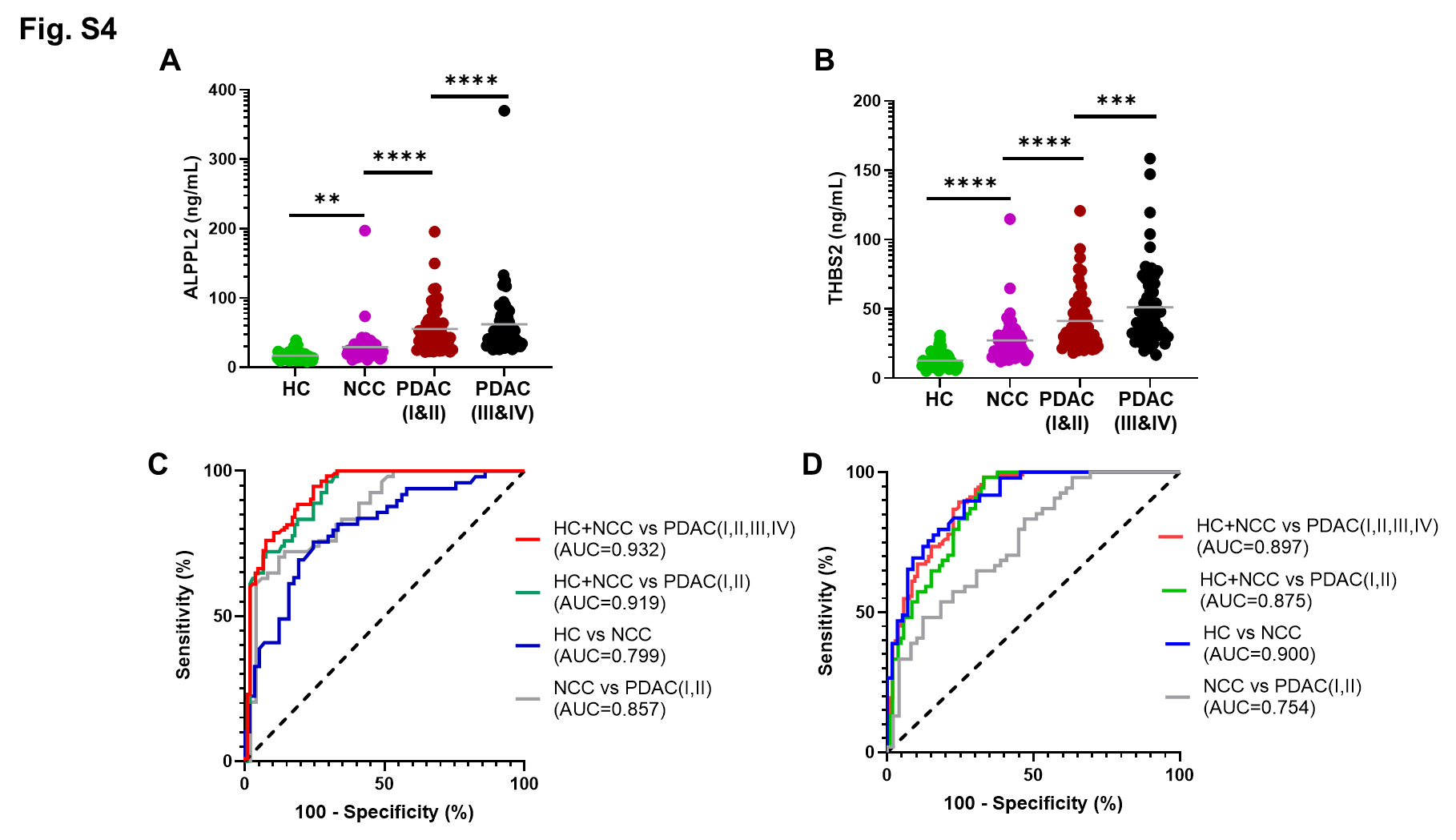
**

**Supplementary Fig. S4**. Performances of serum ALPPL2 and THBS2 measured by ELISA as early detection biomarkers for PDAC. Concentrations of ALPPL2 (A) and THBS2 (B) in serum samples are plotted for individual subjects by groups as listed in Table 1. ROC analysis is used to compare the performances of serum ALPPL2 (C) and THBS2 (D) in discriminating all stages of PDAC from healthy control (HC) and non-cancerous conditions (NCC), early stage (Stage I and II) PDAC from HC and NCC, NCC from HC, and early stage PDAC from NCC. ****P < 0.0001; ***P < 0.001; **P < 0.01.

**
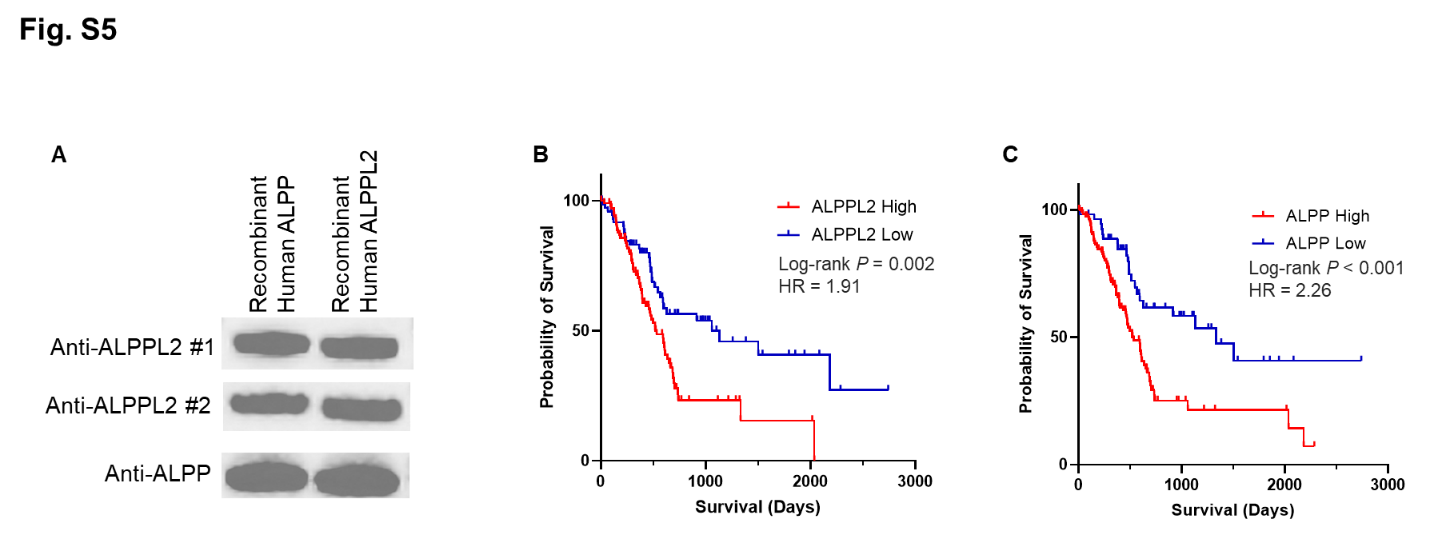
**

**Supplementary Fig. S5.** A) Antibodies against ALPPL2 and ALPP cross react with ALPPL2 and ALPP proteins. Two anti-ALPPL2 antibodies (#1: mouse monoclonal antibody Clone 2B3 from Abnova, Taipei, Taiwan, and #2: Rabbit Polyoclonal antibody from Signalway Antibody, MD, USA) react with both recombinant human ALPPL2 and ALPP and so does the anti-ALPP rabbit monoclonal antibody (Clone JM22-53; Signalway Antibody). Ten nanograms of the recombination proteins, ALPPL2 (Acro Biosystem, San Diego, CA, USA) and ALPP (Acro Biosystem) were loaded to each lane and detected with the anti-ALPPL2 antibody (1:1000) using Western blotting. B and C) Kaplan-Meier survival analysis of CTGA pancreatic adenocarcinoma cohort (CTGA-PAAP) based on mRNA expression of ALPPL2 (B) and ALPP (C). A total of 176 PDAC cases were included in the survival analysis for the ALPPL2 high (N = 75) and low (N = 101) groups or ALPP high (N = 56) and low (N = 120) groups. HR: hazard ratio.
